# Investigating the Impact of Traditional Bullying and Cyberbullying on Suicidal Attempts in Chinese Youth: A Prospective Cohort Study

**DOI:** 10.1155/2024/5860093

**Published:** 2024-11-21

**Authors:** Sihong Li, Xi Ni, Xuerong Luo, Xingyue Jin, Lintong Song, Tianqing Fan, Leyin Zhang, Yanmei Shen

**Affiliations:** Department of Psychiatry, and National Clinical Research Center for Mental Disorders, The Second Xiangya Hospital of Central South University, No.139, Renmin Middle Road, Furong District, Changsha, Hunan, China

**Keywords:** bullying, cyberbullying, mental health, prospective study, suicide

## Abstract

**Aims:** It is unknown whether bullying exposure is independently associated with suicide attempts incidence. This study aims to investigate the association between traditional bullying, cyberbullying, and subsequent risk of suicide attempts among Chinese adolescents.

**Methods:** In this prospective cohort study, 1162 participants (mean age, 12.59, 54.5% male) were recruited from November 2020 to December 2020 in Changsha Hunan Province. In total, 782 of them completed the follow-up in May and June 2021 and were included in the final analysis. Logistic regression was utilized to calculate the odds ratio (OR) with 95% confidence intervals (95% CIs). Gender differences in these associations were further explored by stratified analysis.

**Results:** Adolescents who engaged in bullying perpetration (BP) and experienced cyberbullying victimization (CV) were significantly associated with an increased risk of suicide attempts in the 6-month follow-up even after adjusting for age, ethnicity, single child, depression, anxiety, and stress in the baseline (BP: adjusted OR [aOR] = 3.337, 95% CI: 1.463–7.611, *p*=0.004; CV: aOR = 3.338, 95% CI: 1.468–7.590, *p*=0.004). Furthermore, the association between BP and suicide attempts was found to be statistically significant only among male adolescents (aOR = 6.692, 95% CI: 1.566–28.601, *p*=0.01), while CV was significantly associated with a heightened risk of suicide attempts among female adolescents (aOR = 4.452, 95% CI: 1.684–11.771, *p*=0.003).

**Conclusions:** BP and CV were longitudinally associated with an increased risk of suicide attempts in Chinese youth, and these associations vary across genders.

## 1. Introduction

Adolescence has long recognized suicide as a critical issue concerning public health and globally, it has emerged as one of the primary factors leading to the mortality rate among the youth [1, 2]. Furthermore, it is widely accepted that engaging in suicide attempts serves as the most influential indicator for the occurrence of actual suicide [3]. A previous study showed that male adolescents who attempt suicide are 30 times more likely to end up with committing suicide [4]. Although it is not fatal, suicide attempts have placed heavy financial, social, and psychological strain on individuals, families, and society [5]. Besides, adolescents who attempt suicide are at increased risk for mental health problems [6–8]. Most adolescents who attempt suicide meet the criteria for at least one mental health disorder, including both internal issues (such as depression and anxiety) and external issues (like conduct disorder and aggression), and the strength of these associations typically intensifies as the severity increases [9, 10]. Therefore, it is of great importance to investigate the exposure factors that might lead to suicide attempts.

Adolescence is a period during which bullying is prevalent, manifesting in both traditional forms and cyber forms [1]. Traditional forms of bullying involvement include bullying victimization (BV) and bullying perpetration (BP). BP involves aggressive behavior where individuals in dominant positions cause physical or psychological harm to others [11, 12]. BV refers to the experience of being targeted and harmed by bullying behaviors, while BP involves engaging in bullying behaviors toward others [13]. Furthermore, the expanding influence of the internet has introduced new forms of bullying involvement include cyberbullying victimization (CV) and cyberbullying perpetration (CP) [14]. Cyberbullying, on the other hand, involves deliberate and hostile actions by an individual using electronic communication to continuously target a victim who are unable to easily defend themselves [15]. Notably, the issue of bullying has gained global attention, as evidenced by a meta-analysis of 80 studies, which revealed that traditional BV and perpetration are prevalent at rates of 36% and 35%, respectively. Furthermore, CV and perpetration exhibit prevalence rates of 15.2% and 15.5%, respectively [16]. Specifically, studies indicate that the prevalence of bullying is notably high in middle school age range, a review conducted in Chinese mainland schools has reported that traditional BV rates range from 2% to 66%, while perpetration rates range from 2% to 34% [10]. Additionally, the prevalence of cyberbullying, including both victimization and perpetration, has been reported to range from 14% to 57% and 3% to 35%, respectively [10, 17, 18]. This heightened prevalence can be attributed to several developmental factors. During early adolescence, individuals undergo significant identity formation and are especially sensitive to peer approval. This phase of self-discovery can induce insecurity and vulnerability, leading some to resort to bullying as a means of seeking social acceptance or asserting dominance [19]. Concurrently, the cognitive skills, emotional regulation, and empathy of middle school students are not fully developed, which can lead to impulsive and aggressive behavior, further contributing to the prevalence of bullying [19–21].

Bullying is a well-known risk factor for suicide during adolescence. Research indicated that bullying in early childhood (even at 8 years old) might predict future mental health problems and be associated with subsequent suicide attempts and completed suicides [22]. A positive correlation was discovered in a comprehensive evaluation of 31 studies, highlighting the connection between adolescent engagement in bullying and the occurrence of suicidal behavior [23]. Furthermore, even after accounting for age, gender, ethnicity, and depression, the risk of suicidal behavior remained significantly elevated among individuals who experienced bullying, surpassing that of unaffected youth [24]. Additionally, the examination of gender distinctions in the link between bullying and suicide attempts has yielded inconclusive results. A study in Finnish children reported that girls who are victims of bullying are at higher risk in suicide attempts than boys [22]. A study in China also showed that girls who go through bullying reported suicide attempts more often than boys [25]. However, one study also indicated that there was no significant relationship between bullying and suicidal behavior in boys and girls [26]. As far as we know, few cohort studies have been done to evaluate the gender difference in impact of bullying on suicide attempts. In summary, the purpose of this research was to investigate the connection between exposure to bullying and the occurrence of suicide attempts within a 6-month follow-up period among Chinese middle school students and further explore its gender differences.

## 2. Method

### 2.1. Study Population

A prospective cohort study design was adopted by this study. This study was approved by the ethics committee of the Second Xiangya Hospital of Central South University. Using the cluster sampling method, in Changsha, Hunan Province, 1162 middle school students were recruited from November to December 2020, 78 of them were excluded from this study because they refused to attend. Six months later, from May to June 2021, 848 students agreed to participate in the follow-up survey. Sixty-six were excluded from this study because of present suicide attempts at the time of baseline assessment. Therefore, in the final analysis, 782 students were included. Participants in this study were required to meet the following inclusion criteria: (a) middle school students aged 11–16 years old; (b) no serious medical illness that would prevent them from completing the questionnaire; (c) understand and be able to complete the questionnaire; and (d) willing to sign an informed consent form and participate.

### 2.2. Data Collection and Follow-up

All participants were asked to complete a standardized, self-administered questionnaire regarding socioeconomic status, depression status, anxiety status, stress status, bullying, as well as suicidal attempts in November to December of 2020. And all of them were followed up after 6 months in May and June of 2021 about their suicidal attempts. The professional online survey platform in China named WenJuanXing (www.wjx.cn) is used to create electronic questionnaires, which are distributed through the WeChat platform with the help of school teachers.

### 2.3. Sociodemographic Characteristics

At the baseline, we collected demographic variables from the participants, such as sex, age, ethnicity, as well as family information, including single child.

### 2.4. The Depression Anxiety Stress Scales-21 (DASS-21)

At the start of the study, the DASS-21 were utilized to assess the levels of depression, anxiety, and stress among participants. In this scale, there were a total of 21 items used for clinical evaluation. Specifically, the items measured depression (referred to as DASS-Depression), anxiety (referred to as DASS-Anxiety), and stress (referred to as DASS-Stress). Each of these subscales contained seven items that were rated on a scale ranging from 0 (indicating not experiencing at all) to 3 (indicating experiencing almost every day) [27, 28]. Furthermore, cutoff scores were established for the different subscales, with 10 indicating significant levels of stress, 7 indicating significant levels of anxiety, and 13 indicating significant levels of depression [29]. Previous research has demonstrated that the DASS-21 exhibits strong internal consistency when used with clinical populations [30]. Moreover, studies conducted in China have reported satisfactory internal consistency indices, thereby supporting the cross-cultural validity of this assessment [31].

### 2.5. Bullying

Bullying encompasses both the act of perpetrating bullying and experiencing victimization. Prior to conducting the survey, the definition of both traditional bullying and cyberbullying was elucidated to the participants. The perpetration of traditional bullying was assessed through the inquiry: “Within the last 6 months, have you ever bullied others?” On the other hand, the victimization of traditional bullying was evaluated by asking: “Within the last 6 months, have you experienced any acts of bullying?” The perpetration of cyberbullying was then probed by the question: “Within the past 6 months, have you utilized electronic devices, such as the Internet, mobile phones, Weibo, WeChat, or smartwatches, to bully, mock, or threaten others?” Similarly, the victimization of cyberbullying was gauged by inquiring: “Within the last 6 months, have you encountered instances of being bullied through the Internet, text messages, Weibo, WeChat, mobile phones, smartwatches, or any other electronic device?” The participants indicated the engagement in these behaviors by answering yes or no while responding to the aforementioned queries.

### 2.6. Suicide Attempts

Suicide attempts are indicative of the self-destructive conduct that participants undertake with the intention of ending their lives. To assess suicidal acts, participants were asked the subsequent query designed to require a yes/no answer: “Within the last 6 months, have you made any attempts to take your own life?”.

### 2.7. Statistical Analysis

This study utilized numerical values and proportions to depict categorical variables. Logistic regression analysis was implemented to investigate the correlation between engagement in bullying and the occurrence of suicide attempts. In Model 1, no covariate was adjusted in logistic regression and generated the crude odds ratio (OR), in Model 2, the OR was adjusted by controlling for age, single child, and ethnicity, and in Model 3, the OR was adjusted by controlling for age, single child, ethnicity, anxiety, depression, and stress. Gender differences of these associations were further explored by dividing subjects into male and female groups. Statistical tests were two-tailed. All analyses were conducted with SPSS (Version 26.0; IB.M, Inc., Chicago, IL, USA), and the significance level was set at 0.05.

## 3. Results

**Figure 1:**
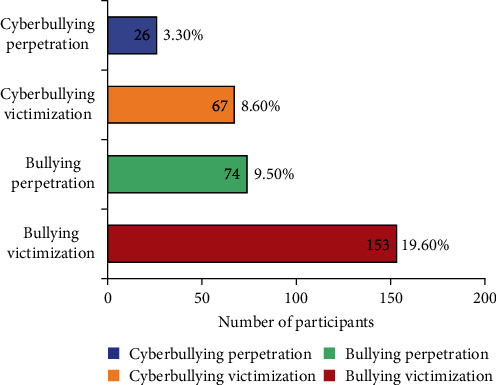
The number of participants involved in different types of bullying.

The baseline characteristics of the study sample are shown in Table 1. A total of 782 participants were included in the analysis, with an average age of 12.59 years (standard deviation [SD], 0.64). The majority of participants were male (54.5%), Han ethnic (95.0%), and not the single child in the family (59.0%). Most participants did not have a history of depression (69.2%), anxiety (53.1%), or stress (79.0%). Furthermore, as shown in Figure 1, around 9.5% and 3.3% of participants had been a perpetrator of traditional bullying and cyberbullying. Yet the proportion of victims is higher. Around 19.6% and 8.6% of the participants had been a victim of traditional bullying and cyberbullying.

**Figure 2:**
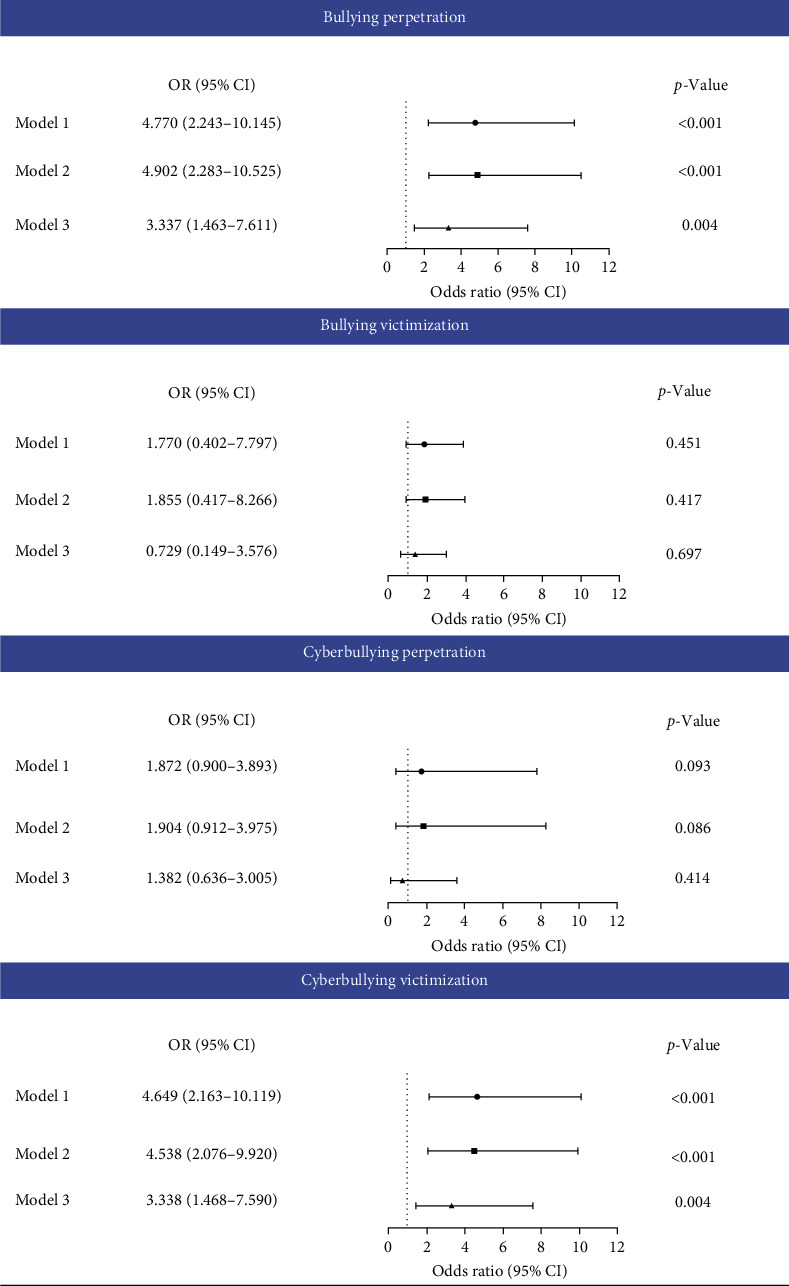
The odds ratio (OR) and 95% confidence intervals (95% CIs) of the associations between bullying involvement and suicide attempts. Model 1: unadjusted. Model 2: adjusted for age, ethnicity, and single child. Model 3: adjusted for age, ethnicity, single child, and depression, anxiety, and stress at baseline.

As shown in Figure 2, the results indicated that adolescents who engaged in BP and experienced CV were significantly associated with an increased risk of suicide attempts in half a year, even after adjusting for age, ethnicity, single child, depression, anxiety, and stress in the baseline. Adolescents who engaged in BP reported a higher risk of suicide attempts (BP adjusted OR [aOR] = 3.337, 95% confidence interval [CI]: 1.463–7.611, *p*=0.004; CV: aOR = 3.338, 95% CI: 1.468–7.590, *p*=0.004). However, the association between BV, CP, and suicide attempts was nonsignificant.

**Figure 3:**
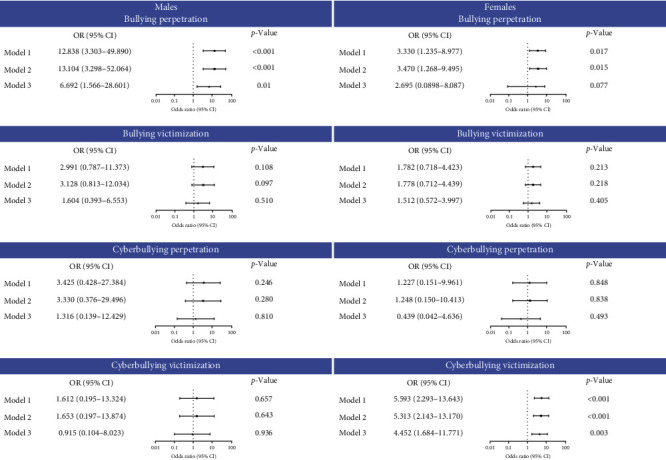
The odds ratio (OR) and 95% confidence intervals (95% CIs) of the associations between bullying involvement and suicide attempts stratified by gender. Model 1: unadjusted. Model 2: adjusted for age, ethnicity, and single child. Model 3: adjusted for age, ethnicity, single child, and depression, anxiety, and stress at baseline.

Figure 3 shows the relationships between bullying involvement exposure and the risk of suicide attempts in the males and females using three logistic regression models adjusted for different covariates. As for male students who engaged in BP, the crude OR of suicidal attempts (Model 1) was 12.838 (95% CI: 3.303–49.890). When adjusted for demographic characteristics such as age, ethnicity, single child (Model 2), and in addition to anxiety, depression, and stress (Model 3), the risk of suicide attempts was still significantly higher, with the OR of Model 2 is 13.104 (95% CI: 3.298–52.064) and Model 3 is 6.692 (95%CI: 1.566–28.601).

However, in female students who engaged in BP, the OR in Model 1 was 3.330 (95% CI: 1.235–8.977), and the OR in Model 2 was 3.470 (95% CI: 1.268–9.495). In Model 3, when adding an adjustment for anxiety, depression, and stress, BP is insignificantly associated with suicide attempts in female students. Specifically, in female students, CV was significantly associated with suicide attempts in all three models, with OR in Model 1 was 5.593 (95% CI: 2.293–13.643), in Model 2 was 5.313 (95% CI: 2.143–13.170), as well as in Model 3 was 4.452 (95% CI: 1.684–11.771).

## 4. Discussion

In this study, longitudinal associations between BP and BV, CP and CV, and adolescent suicide attempts were examined over a follow-up of 6 months. Both engaging in BP and suffering from CV within the past 6 months were significantly associated with an increased risk of adolescent suicide attempts even after taking variable confounders into consideration. Furthermore, gender differences were found. Specifically, the association between engaging in BP and adolescent suicide attempts was found to be statistically significant exclusively among male adolescents. Conversely, experiencing CV was significantly associated with a heightened risk of suicide attempts among female adolescents.

### 4.1. Prevalence of Bullying

In this study, the prevalence of BV and perpetration was 19.6% and 9.5%, respectively, while for cyberbullying, it was 8.6% and 3.3%. This suggests that bullying is common among middle school-aged students in China, highlighting the need for measures to reinforce the prevention of bullying. Consistent with a prior systematic review among Chinese youth [32], the current study indicated that traditional bullying is more common than cyberbullying, and victimization rates are higher than perpetration in both forms. The higher prevalence of traditional bullying compared to cyberbullying can be attributed to several factors. First, traditional bullying typically occurs in school environments where students interact daily in classrooms, hallways, and playgrounds. The tangible presence of these physical settings facilitates traditional bullying more readily than cyberbullying. Cyberbullying requires access to digital platforms, while technology use is increasing, middle school students may have limited access to digital devices or online platforms compared to older age groups. For instance, a study showed the average screen time for Chinese middle and high school students was 2.65 ± 3.39 and 3.52 ± 2.7 h/day [33]. This limited access may lead to lower rates of cyberbullying compared to traditional bullying. Also, middle school students are in a crucial stage of developing their social identities and navigating peer relationships. Traditional bullying may be more prevalent during this time as students seek to establish dominance and affirm their social status [34].

The higher prevalence of victimization compared to perpetration among middle school students can be attributed to several factors. First, bullying often involves a power imbalance, where middle school students are more likely to be victims due to their relatively vulnerable position [35]. Besides, cultural norms in China emphasize harmony and group cohesion while discouraging aggressive behaviors [36]. Consequently, reports of bullying may be underreported, and the prevalence of bullying may be underestimated due to cultural stigmas.

### 4.2. Association of BV and BP With Suicidal Attempt

In line with previous studies [37], our research confirms that BP is longitudinally associated with increased suicide attempts. Suicide theories, which integrate psychological, social, cognitive, emotional, and biological factors, help explain this relationship [38]. The Integrated Motivational-Volitional Model (The IMV Model) [39] and the Interpersonal Psychological Theory of Suicide [40] both elucidate the link between BP and suicide attempts, emphasizing the shared factors such as emotional distress, social rejection, and feelings of isolation. These theories suggest that BP intensifies despair and hopelessness through negative emotions and guilt, while social rejection and failure further exacerbate these feelings, leading to greater helplessness. Moreover, both theories recognize that adolescents who bully others may experience perceived burdensomeness and thwarted belongingness, amplifying their sense of isolation. The IMV Model additionally highlights how persistent distress and impulsivity associated with bullying can reinforce suicidal intent, whereas the Interpersonal Theory underscores the role of perceived burdensomeness and the capability for suicide. Together, these factors significantly elevate the risk of suicide attempts among those involved in BP.

Contrary to previous research [41, 42], increased risk for suicide attempts was not observed in association with BV in this study. A possible reason could be variations in how bullying is defined across different studies. For instance, some studies define being bullied as experiencing at least three times of bullying within the past month [43]. The reported frequency and severity of being bullied in this study might have been relatively mild, and the time frame was limited to past 6 months, which might potentially be insufficient to lead to the consequences of suicide attempts. Moreover, although numerous studies suggested a positive association between BV and suicidal behavior, the majority of them are cross-sectional in design.

### 4.3. Association of CV and CP With Suicidal Attempt

In recent years, appreciation of the role of cyberbullying in suicide has burgeoned. Consistent with the previous study [44–47], our study found that CV was significantly associated with a higher risk of suicidality among adolescents. Prior studies suggest that the negative impacts of CV might surpass those of traditional BV [48]. One potential explanation is that online anonymity makes it difficult for victims to quickly identify perpetrators and retaliate, while the viral nature of the Internet amplifies the exposure of victims' personal information [49]. Furthermore, adolescent suicides exhibit clustering tendencies. Young people susceptible to suicidal behavior tend to associate with others at risk, and the spread of suicidal thoughts might facilitate further suicides [50]. According to the social contagion theory, the Internet, as an intermediary, plays a pivotal role in this mechanism [51]. Adolescents experiencing CV may engage in mutual communication online and access suicide-related information rapidly beyond traditional geographical boundaries.

Contrary to the association observed with CV, no longitudinal evidence was found linking CP to suicidal attempts [37]. This finding aligns with a large-scale study conducted in the United States, which demonstrated that while CV is associated with a higher risk of suicidality in early adolescence, CP is not [52]. One possible explanation for this discrepancy is that perpetrators of cyberbullying may not directly witness the immediate harm caused to their victims, potentially leading to reduced feelings of guilt and responsibility. Consequently, the negative psychological impact of engaging in cyberbullying may be less pronounced in perpetrators compared to victims.

### 4.4. Gender Difference in the Association of Bullying and Suicidal Attempt

To our knowledge, this was the first prospective cohort study to elucidate gender differences in the association between bullying and suicide attempts among adolescents. Gender difference analysis reveals that the association between CV and increased risk of suicide attempts is significant only among females. In contrast, BP is only significantly associated with suicide attempts among males. This could be attributed to the fact that males and females engaged in distinct forms of subtypes. Males are more prone to engage in physical and verbal bullying, while females tend to be engaged in relational bullying [53]. A recent study indicated that the risk factors for suicide attempts vary between male and female adolescents, depressive symptoms and interpersonal issues are specific risk factors for suicide attempts in females, while disruptive behavior and conduct problems are specific risk factors for suicide attempts in males [54]. Previous research indicated that females who experience CV are at a higher risk of developing depression and suicide ideation compared to males [55, 56]. This difference may be related to rumination. Rumination is a form of repetitive negative thinking, which involves repeatedly and negatively focusing on discomfort and distress rather than attempting to solve the problem [57]. A strong mediator, the onset of the gender differences in rumination occurred at 12 years [58]. When experiencing CV, the risk of depression is higher in females due to the mediating effect of rumination compared to males [59]. Neuroendocrine mechanisms may play a role in this association, and changes in hormone levels during adolescence can influence the development of brain circuits involved in regulating social cognition, emotion regulation, and impulse control. The amygdala, hippocampus, and anterior cingulate cortex (ACC) form a network for detecting social salience, the amygdala and medial prefrontal cortex (mPFC) form a crucial emotion regulation circuit, and the circuits formed by striatum, frontal regions, and the orbitofrontal cortex (OFC) govern reward valuation and impulse control [60]. Dysfunction in these brain regions is involved in suicide [61]. Notably, several of these brain regions already exhibit gender-based differences during adolescence, for example, boys were reported to have larger amygdala and hippocampal volumes, increased hippocampal, and striatal variance compared to girls [62]. When facing adversity, the fluctuations of gonadal hormones, including progesterone, estrogens, and testosterone, may lead to different dysfunctions in males and females in brain circuits, such as amygdala connectivity with PFC and hippocampal connectivity with PFC [63].

Several limitations need to be considered. First, an important limitation of observational studies is the challenge of establishing causality and addressing residual confounding. Second, although a significant number of relevant confounding factors were controlled, we cannot disregard the potential impact of unmeasured confounders on these results. Third, since both bullying involvement and suicide attempts are based on self-report questionnaires, potential biases may be introduced. Fourth, participants who were not involved in bullying at baseline might encounter relevant experiences during the follow-up. Fifth, different forms of bullying might overlap. A prior study indicated that cyber victimization coincided with traditional victimization in 85.2% of cases [64]. Research indicates that being a victim of both cyberbullying and traditional bullying poses a higher risk of suicide attempts compared to being a victim of either type alone.

## 5. Conclusions

This study supports a prospective association between BP, CV, and suicide attempts among adolescents. Furthermore, gender differences were observed in these associations. The association between BP and adolescent suicide attempts was found to be statistically significant among male adolescents, while CV was significantly associated with a heightened risk of suicide attempts among female adolescents. These findings indicated that both BP and CV might be important risk factors for adolescents' suicide attempts, with varying impacts on males and females. This suggests that interventions focusing on school bullying, internet bullying, and peer relationships could help in reducing and preventing adolescent suicide. Future research is required to determine causality and underlying mechanisms between bullying and suicidal behavior.

## Figures and Tables

**Table 1:** Demographic characteristics of participants (*n* = 782).

Characteristic	Number of participants	Percent (%)
Age, mean (SD)	12.59 (0.64)	—
Gender		
Male	426	54.5
Female	356	45.5
Ethnic group		
Han	743	95.0
Others	39	5.0
Single child		
No	462	59.0
Yes	319	41.0
Depression		
No	541	69.2
Yes	241	30.8
Anxiety		
No	415	53.1
Yes	367	46.9
Stress		
No	618	79.0
Yes	164	21.0
Suicide attempts in half a year		
No	746	95.4
Yes	36	4.6
BP		
No	708	90.5
Yes	74	9.5
BV		
No	629	80.4
Yes	153	19.6
CP		
No	756	96.7
Yes	26	3.3
CV		
No	715	91.4
Yes	67	8.6

Abbreviations: BP, bullying perpetration; BV, bullying victimization; CP, cyberbullying perpetration; CV, cyberbullying victimization; SD, standard deviation.

## Data Availability

The data that support the findings of this study are available on request from the corresponding author. The data are not publicly available due to privacy or ethical restrictions.

## References

[1] Silverman M. M., Berman A. L., Sanddal N. D., O’Carroll P. W., Joiner T. E. (2007). Rebuilding the Tower of Babel: A Revised Nomenclature for the Study of Suicide and Suicidal Behaviors. Part 2: Suicide-Related Ideations, Communications, and Behaviors. *Suicide and Life-Threatening Behaviour*.

[2] Abio A., Owusu P. N., Posti J. P. (2022). Cross-National Examination of Adolescent Suicidal Behavior: A Pooled and Multi-Level Analysis of 193,484 Students from 53 LMIC Countries. *Social Psychiatry and Psychiatric Epidemiology*.

[3] Xu Y., Wang C., Shi M. (2018). Identifying Chinese Adolescents With a High Suicide Attempt Risk. *Psychiatry Research*.

[4] Shaffer D., Gould M. S., Fisher P. (1996). Psychiatric Diagnosis in Child and Adolescent Suicide. *Archives of General Psychiatry*.

[5] Koyanagi A., Oh H., Carvalho A. F. (2019). Bullying Victimization and Suicide Attempt Among Adolescents Aged 12–15 Years From 48 Countries. *Journal of the American Academy of Child & Adolescent Psychiatry*.

[6] Shan J. C., Chen I. M., Lin P. H. (2022). Associations Between Lifetime Mental Disorders and Suicidal Behaviors: Findings From the Taiwan Psychiatry Morbidity Survey. *Social Psychiatry and Psychiatric Epidemiology*.

[7] Gili M., Castellví P., Vives M. (2019). Mental Disorders as Risk Factors for Suicidal Behavior in Young People: A Meta-Analysis and Systematic Review of Longitudinal Studies. *Journal of Affective Disorders*.

[8] Orri M., Scardera S., Perret L. C. (2020). Mental Health Problems and Risk of Suicidal Ideation and Attempts in Adolescents. *Pediatrics*.

[9] Goldston D. B., Daniel S. S., Erkanli A. (2009). Psychiatric Diagnoses as Contemporaneous Risk Factors for Suicide Attempts Among Adolescents and Young Adults: Developmental Changes. *Journal of Consulting and Clinical Psychology*.

[10] Chan H. C., Wong D. S. W. (2015). Traditional School Bullying and Cyberbullying in Chinese Societies: Prevalence and a Review of the Whole-School Intervention Approach. *Aggression and Violent Behavior*.

[11] Olweus D. (2013). School Bullying: Development and Some Important Challenges. *Annual Review of Clinical Psychology*.

[12] Ybarra M. L., Espelage D. L., Valido A., Hong J. S., Prescott T. L. (2019). Perceptions of Middle School Youth about School Bullying. *Journal of Adolescence*.

[13] Espelage D. L., De La Rue L. (2011). School Bullying: Its Nature and Ecology. *International Journal of Adolescent Medicine and Health*.

[14] Giumetti G. W., Kowalski R. M. (2022). Cyberbullying via Social Media and Well-Being. *Current Opinion in Psychology*.

[15] Smith P. K., Mahdavi J., Carvalho M., Fisher S., Russell S., Tippett N. (2008). Cyberbullying: Its Nature and Impact in Secondary School Pupils. *Journal of Child Psychology and Psychiatry*.

[16] Modecki K. L., Minchin J., Harbaugh A. G., Guerra N. G., Runions K. C. (2014). Bullying Prevalence Across Contexts: A Meta-Analysis Measuring Cyber and Traditional Bullying. *Journal of Adolescent Health*.

[17] Lam L. T., Cheng Z. H., Liu X. M. (2013). Violent Online Games Exposure and Cyberbullying/Victimization Among Adolescents. *Cyberpsychology, Behavior, and Social Networking*.

[18] Li Q. (2008). A Cross-Cultural Comparison of Adolescents’ Experience Related to Cyberbullying. *Educational Research*.

[19] Thomas H. J., Connor J. P., Scott J. G. (2018). Why Do Children and Adolescents Bully Their Peers? A Critical Review of Key Theoretical Frameworks. *Social Psychiatry and Psychiatric Epidemiology*.

[20] Sutton J., Smith P. K., Swettenham J. (1999). Bullying and Theory of Mind: A Critique of the Social Skills Deficit View of Anti-Social Behaviour. *Social Development*.

[21] Malin Y., Gumpel T. P. (2023). Dispositional Mindfulness Plays a Major Role in Adolescents’ Active and Passive Responding to Bully-Victim Dynamics. *Aggressive Behavior*.

[22] Klomek A. B., Sourander A., Niemelä S. (2009). Childhood Bullying Behaviors as a Risk for Suicide Attempts and Completed Suicides: A Population-Based Birth Cohort Study. *Journal of the American Academy of Child & Adolescent Psychiatry*.

[23] Klomek A. B., Sourander A., Gould M. (2010). The Association of Suicide and Bullying in Childhood to Young Adulthood: A Review of Cross-Sectional and Longitudinal Research Findings. *The Canadian Journal of Psychiatry*.

[24] Kaminski J. W., Fang X. (2009). Victimization by Peers and Adolescent Suicide in Three US Samples. *The Journal of Pediatrics*.

[25] Yang T., Guo L., Hong F., Wang Z., Yu Y., Lu C. (2020). Association Between Bullying and Suicidal Behavior Among Chinese Adolescents: An Analysis of Gender Differences. *Psychology Research and Behavior Management*.

[26] Kim Y. S., Leventhal B. L., Koh Y.-J., Boyce W. T. (2009). Bullying Increased Suicide Risk: Prospective Study of Korean Adolescents. *Archives of Suicide Research*.

[27] Antony M. M., Cox B. J., Enns M. W., Bieling P. J., Swinson R. P. (1998). Psychometric Properties of the 42-Item and 21-Item Versions of the Depression Anxiety Stress Scales in Clinical Groups and a Community Sample. *Psychological Assessment*.

[28] Babbitt R. L., Edlen L., Antony M. M. (1998). Psychometric Properties of the 42-Item and 21-Item Versions of the Depression Anxiety and Nezin.

[29] Lovibond P. F., Lovibond S. H. (1995). The Structure of Negative Emotional States: Comparison of the Depression Anxiety Stress Scales (DASS) with the Beck Depression and Anxiety Inventories. *Behaviour Research and Therapy*.

[30] Bibi A., Lin M., Zhang X. C., Margraf J. (2020). Psychometric Properties and Measurement Invariance of Depression, Anxiety and Stress Scales (DASS-21) across Cultures. *International Journal of Psychology*.

[31] Wang K., Shi H., Geng F. (2016). Cross-Cultural Validation of the Depression Anxiety Stress Scale-21 in China. *Psychological Assessment*.

[32] Xing J., Peng M., Deng Z., Chan K. L., Chang Q., Ho R. T. H. (2023). The Prevalence of Bullying Victimization and Perpetration Among the School-Aged Population in Chinese Communities: A Systematic Review and Meta-Analysis. *Trauma, Violence & Abuse*.

[33] Liu S., Lan Y., Chen B., He G., Jia Y. (2022). Smartphone Use Time and Total Screen Time Among Students Aged 10-19 and the Effects on Academic Stress: A Large Longitudinal Cohort Study in Shanghai, China. *Frontiers in Public Health*.

[34] Pan B., Zhang L., Ji L., Garandeau C. F., Salmivalli C., Zhang W. (2020). Classroom Status Hierarchy Moderates the Association Between Social Dominance Goals and Bullying Behavior in Middle Childhood and Early Adolescence. *Journal of Youth and Adolescence*.

[35] Chaux E., Castellanos M. (2015). Money and Age in Schools: Bullying and Power Imbalances. *Aggressive Behavior*.

[36] Chen X., Cen G., Li D., He Y. (2005). Social Functioning and Adjustment in Chinese Children: The Imprint of Historical Time. *Child Development*.

[37] Benatov J., Klomek A. B., Chen-Gal S. (2022). Bullying Perpetration and Victimization Associations to Suicide Behavior: A Longitudinal Study. *European Child & Adolescent Psychiatry*.

[38] Díaz-Oliván I., Porras-Segovia A., Barrigón M. L., Jiménez-Muñoz L., Baca-García E. (2021). Theoretical Models of Suicidal Behaviour: A Systematic Review and Narrative Synthesis. *European Journal of Psychiatry*.

[39] O’Connor R. C., Kirtley O. J. (2018). The Integrated Motivational-Volitional Model of Suicidal Behaviour. *Philosophical Transactions of the Royal Society B: Biological Sciences*.

[40] Joiner T. E. (2009). Main Predictions of the Interpersonal-Psychological Theory of Suicidal Behavior: Empirical Tests in Two Samples of Young Adults. *Journal of Abnormal Psychology*.

[41] Peng C., Hu W., Yuan S. (2020). Suicidal Ideation, and Suicide Attempts in Chinese Adolescents Involved in Different Sub-Types of Bullying: A Cross-Sectional Study. *Frontiers in Psychiatry*.

[42] Sampasa-Kanyinga H., Lalande K., Colman I. (2020). Cyberbullying Victimisation and Internalising and Externalising Problems Among Adolescents: The Moderating Role of Parent-Child Relationship and Child’s Sex. *Epidemiology and Psychiatric Sciences*.

[43] Goldbach J. T., Sterzing P. R., Stuart M. J. (2018). Challenging Conventions of Bullying Thresholds: Exploring Differences between Low and High Levels of Bully-Only, Victim-Only, and Bully-Victim Roles. *Journal of Youth and Adolescence*.

[44] Maurya C., Muhammad T., Dhillon P., Maurya P. (2022). The Effects of Cyberbullying Victimization on Depression and Suicidal Ideation Among Adolescents and Young Adults: A Three Year Cohort Study From India. *BMC Psychiatry*.

[45] Peprah P., Oduro M. S., Okwei R., Adu C., Asiamah-Asare B. Y., Agyemang-Duah W. (2023). Cyberbullying Victimization and Suicidal Ideation Among in-School Adolescents in Three Countries: Implications for Prevention and Intervention. *BMC Psychiatry*.

[46] Nguyen H. T. L., Nakamura K., Seino K., Vo V. T. (2020). Relationships among Cyberbullying, Parental Attitudes, Self-Harm and Suicidal Behavior among Adolescents: Results From a School-Based Survey in Vietnam. *BMC Public Health*.

[47] Kee D. M. H., Anwar A., Vranjes I. (2024). Cyberbullying Victimization and Suicide Ideation: The Mediating Role of Psychological Distress Among Malaysian Youth. *Computers in Human Behavior*.

[48] Ossa F. C., Jantzer V., Neumayer F., Eppelmann L., Resch F., Kaess M. (2023). Cyberbullying and School Bullying Are Related to Additive Adverse Effects among Adolescents. *Psychopathology*.

[49] Kowalski R. M., Giumetti G. W., Schroeder A. N., Lattanner M. R. (2014). Bullying in the Digital Age: A Critical Review and Meta-Analysis of Cyberbullying Research Among Youth. *Psychological Bulletin*.

[50] Hawton K., Hill N. T. M., Gould M., John A., Lascelles K., Robinson J. (2020). Clustering of Suicides in Children and Adolescents. *The Lancet Child & Adolescent Health*.

[51] Haw C., Hawton K., Niedzwiedz C., Platt S. (2013). Suicide Clusters: A Review of Risk Factors and Mechanisms. *Suicide and Life-Threatening Behavior*.

[52] Arnon S., Brunstein Klomek A., Visoki E. (2022). Association of Cyberbullying Experiences and Perpetration With Suicidality in Early Adolescence. *JAMA Network Open*.

[53] Wang J., Iannotti R. J., Nansel T. R. (2009). School Bullying Among Adolescents in the United States: Physical, Verbal, Relational, and Cyber. *Journal of Adolescent Health*.

[54] Miranda-Mendizabal A., Castellví P., Parés-Badell O. (2019). Gender Differences in Suicidal Behavior in Adolescents and Young Adults: Systematic Review and Meta-Analysis of Longitudinal Studies. *International Journal of Public Health*.

[55] Hu Y., Bai Y., Pan Y., Li S. (2021). Cyberbullying Victimization and Depression Among Adolescents: A Meta-Analysis. *Psychiatry Research*.

[56] Kim S., Kimber M., Boyle M. H., Georgiades K. (2019). Sex Differences in the Association Between Cyberbullying Victimization and Mental Health, Substance Use, and Suicidal Ideation in Adolescents. *The Canadian Journal of Psychiatry*.

[57] Shaw Z. A., Hilt L. M., Starr L. R. (2019). The Developmental Origins of Ruminative Response Style: An Integrative Review. *Clinical Psychology Review*.

[58] Jose P. E., Brown I. (2008). When Does the Gender Difference in Rumination Begin? Gender and Age Differences in the Use of Rumination by Adolescents. *Journal of Youth and Adolescence*.

[59] Espinosa F., Martin-Romero N., Sanchez-Lopez A. (2022). Repetitive Negative Thinking Processes Account for Gender Differences in Depression and Anxiety During Adolescence. *International Journal of Cognitive Therapy*.

[60] McEwen B. S., Milner T. A. (2017). Understanding the Broad Influence of Sex Hormones and Sex Differences in the Brain. *Journal of Neuroscience Research*.

[61] Kamimura H., Matsuoka T., Okai H., Shimizu N., Harada S., Matsuo K. (2022). The Associations Between Suicide-Related Behaviors, Prefrontal Dysfunction in Emotional Cognition, and Personality Traits in Mood Disorders. *Scientific Reports*.

[62] Kaczkurkin A. N., Raznahan A., Satterthwaite T. D. (2019). Sex Differences in the Developing Brain: Insights From Multimodal Neuroimaging. *Neuropsychopharmacology*.

[63] Ho T. C., Gifuni A. J., Gotlib I. H. (2022). Psychobiological Risk Factors for Suicidal Thoughts and Behaviors in Adolescence: A Consideration of the Role of Puberty. *Molecular Psychiatry*.

[64] Wolke D., Lee K., Guy A. (2017). Cyberbullying: A Storm in a Teacup?. *European Child & Adolescent Psychiatry*.

